# Supplementary material for: Novel Non-Metal Cation (NMC) Pentaborate Salts of Some Amino Acids
Source: Molecules. 2019 Jul 31;24(15):2790. doi: 10.3390/molecules24152790 (PMC6695838; doi:10.3390/molecules24152790)
Supplement: Supplementary file 1 [file molecules-24-02790-s001.pdf]

SUPPLEMENTARY FILE

Novel Non-Metal Cations (NMCs) Pentaborate Salts with Some Aminoacids

Ümit SIZIR[a], Ömer YURDAKUL[a], Dursun Ali KÖSE[a] and Fatih AKKURT[b]

SI Table 1. ^{11}B-NMR analysis values of pentaborate salts containing organic compound as a complementary cation.

Pentaborate salts	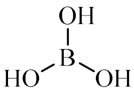 Boric acid	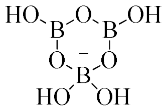 Triborate anion	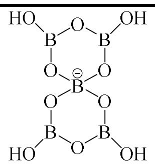 Pentaborate anion
$[C_5H_{11}NO_2][B_5O_6(OH)_4]H_2O$ $C_5H_{17}B_5NO_{13}$	19.37	12.53	1.17
$[C_6H_{13}NO_2][B_5O_6(OH)_4]1/2H_2O$ $C_6H_{18}B_5NO_{12,5}$	17.73	13.06	1.08
$[C_6H_{13}NO_2][B_5O_6(OH)_4]H_2O$ $C_6H_{29}B_5NO_{13}$	18.63	12.94	1.07
$[C_4H_9NO_3][B_5O_6(OH)_4]H_2O$ $C_4H_{15}B_5NO_{14}$	18.84	13.32	1.00

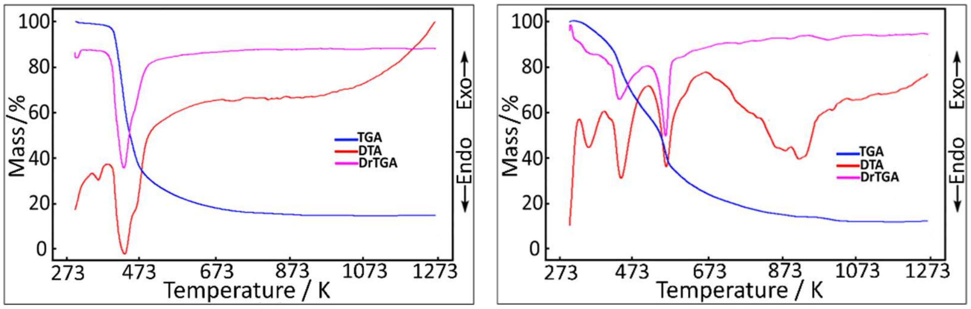

(a) (b)

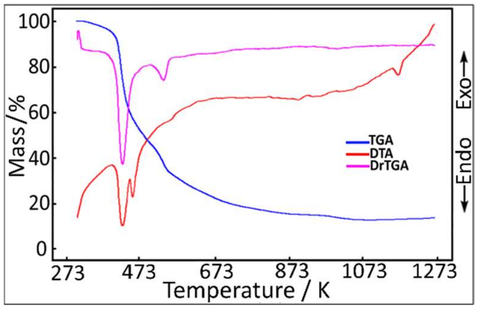

(c)

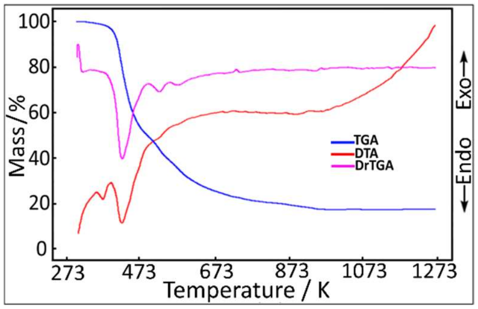

(d)

SI Figure 1. Thermal analysis curves of aminoacid pentaborate salts. **(a)** valine pentaborate, **(b)** leucine pentaborate, **(c)** isoleucine pentaborate, **(d)** threonine pentaborate.

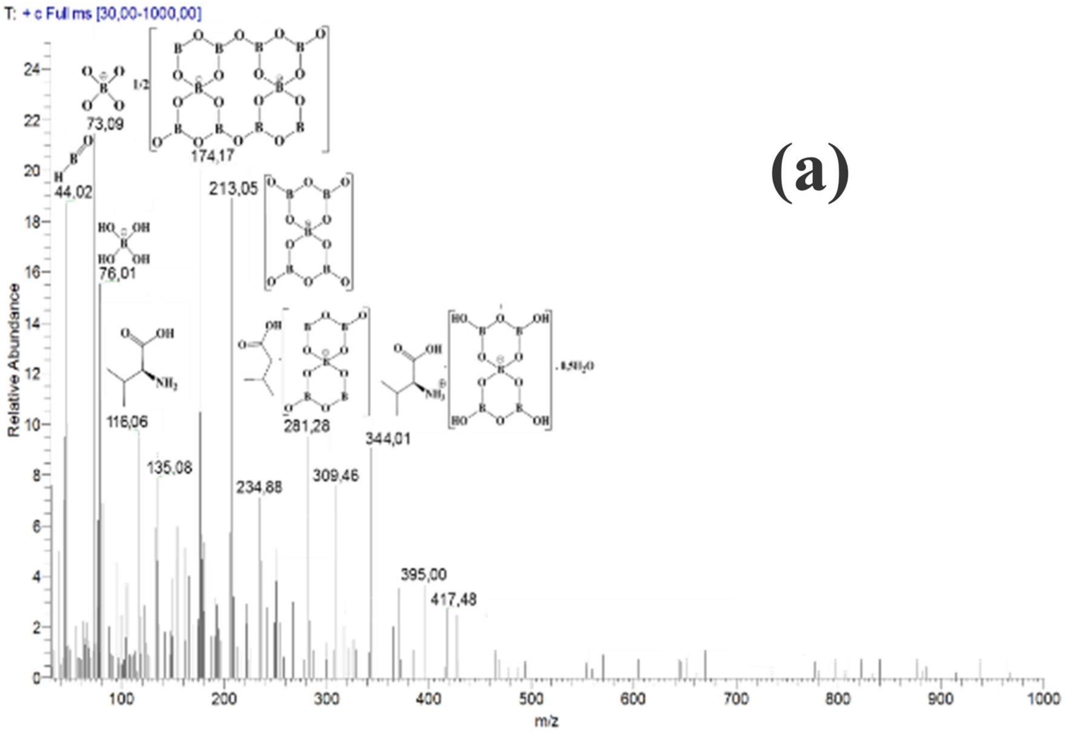
T: + c Full ms [30,00-1000,00]
(a)
Relative Abundance
m/z
44,02
73,09
174,17
213,05
76,01
116,06
135,08
234,88
281,28
309,46
344,01
395,00
417,48

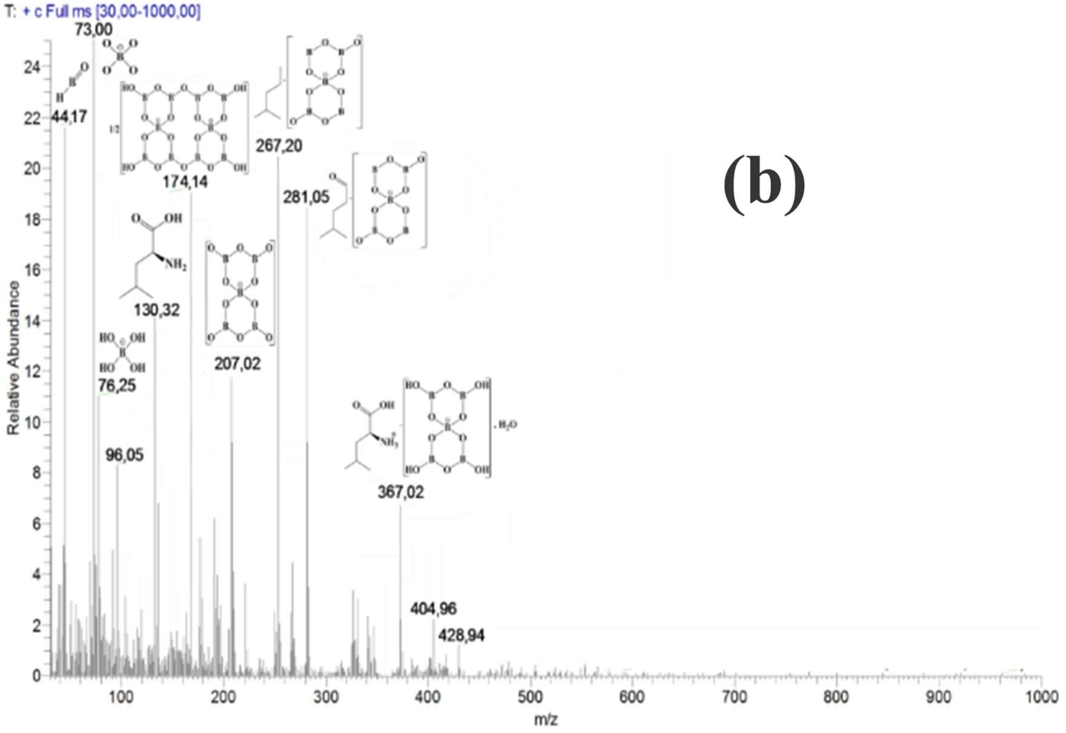
T: + c Full ms [30,00-1000,00]
(b)
Relative Abundance
m/z
73,00
44,17
174,14
267,20
281,05
130,32
76,25
207,02
96,05
367,02
404,96
428,94

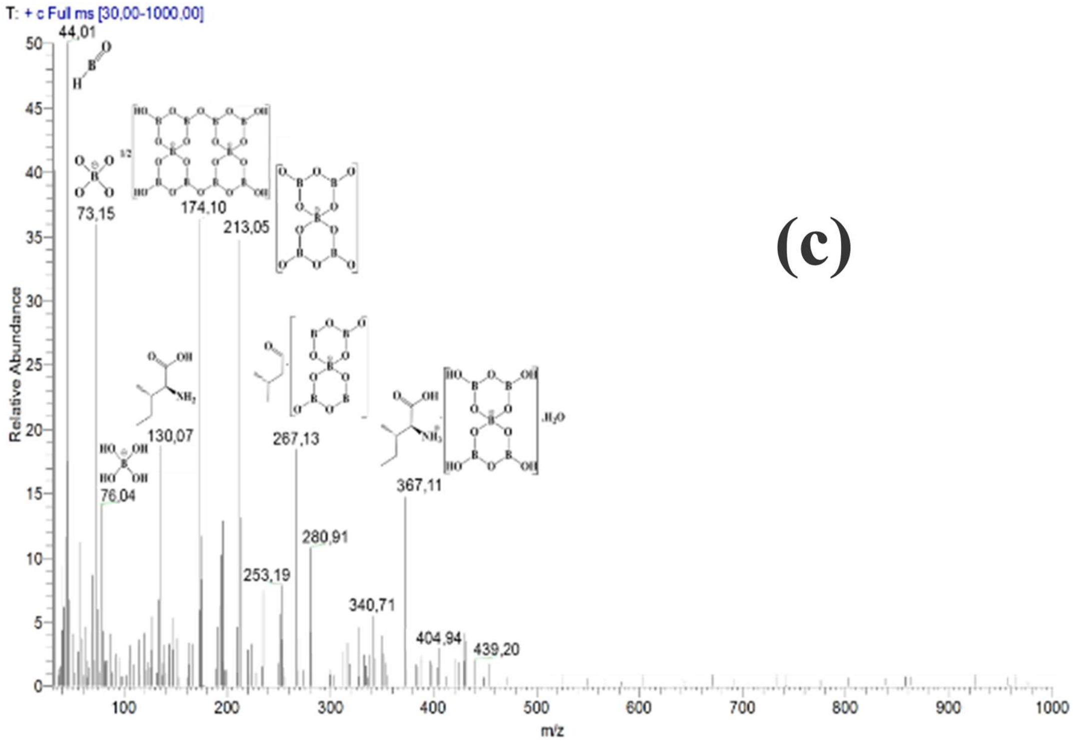

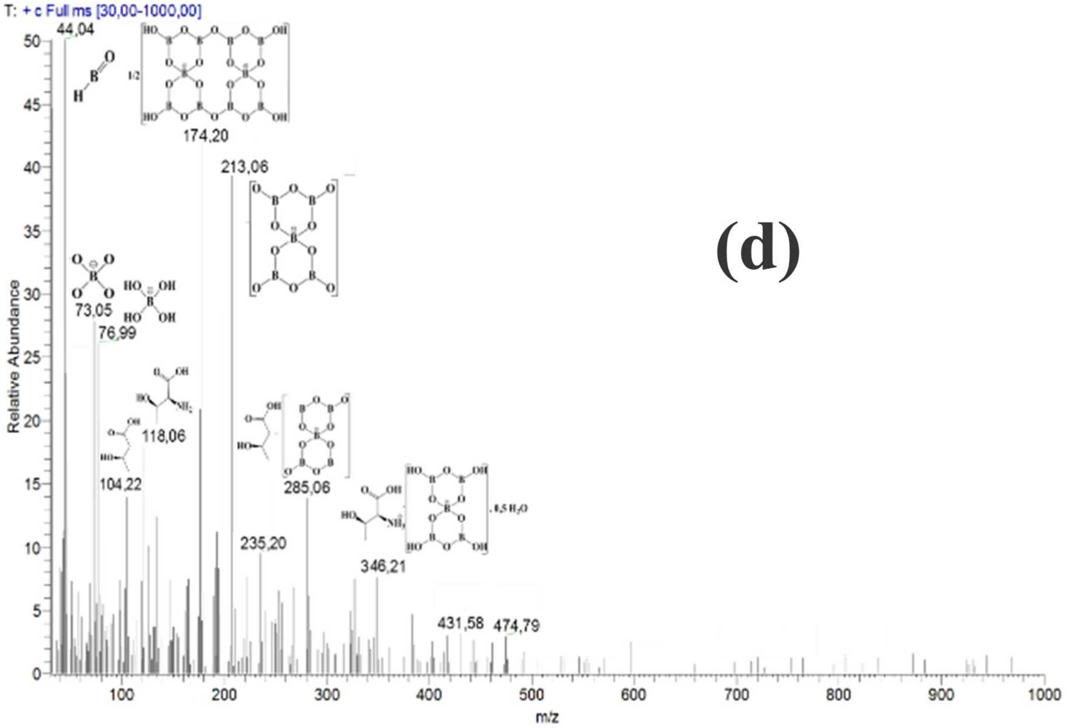

SI Figure 2. Mass spectroscopy analysis of aminoacid pentaborate salts. **(a)** valine pentaborate, **(b)** leucine pentaborate, **(c)** isoleucine pentaborate, **(d)** threonine pentaborate.
